# Consistency of muscle fibers directionality in human thigh derived from diffusion-weighted MRI

**DOI:** 10.1088/1361-6560/acf10c

**Published:** 2023-09-01

**Authors:** Nadya Shusharina, Christopher Nguyen

**Affiliations:** 1 Division of Radiation Biophysics, Department of Radiation Oncology, Massachusetts General Hospital, Boston, MA 02114, United States of America; 2 Harvard Medical School, Boston, MA 02115, United States of America; 3 Cardiovascular Innovation Research Center, Heart, Vascular, and Thoracic Institute, Cleveland Clinic, Cleveland, OH, United States of America

**Keywords:** diffusion-weighted magnetic resonance imaging, skeletal muscle fibers, clinical target volume, Eikonal equation, diffusion tensor

## Abstract

*Objective.* Diffusion-weighted MR imaging (DW-MRI) is known to quantify muscle fiber directionality and thus may be useful for radiotherapy target definition in sarcomas. Here, we investigate the variability of tissue anisotropy derived from diffusion tensor (DT) in the human thigh to establish the baseline parameters and protocols for DW-MRI acquisition for future studies in sarcoma patients. *Approach.* We recruited ten healthy volunteers to acquire diffusion-weighted MR images of the left and right thigh. DW-MRI data were used to reconstruct DT eigenvectors within each individual thigh muscle. Deviations of the principal eigenvector from its mean were calculated for different experimental conditions. *Main results.* Within the majority of muscles in most subjects, the mode of the histogram of the angular deviation of the principal eigenvector of the water DT from its muscle-averaged value did not exceed 20°. On average for all subjects, the mode ranged from 15° to 24°. Deviations much larger than 20° were observed in muscles far from the RF coil, including cases with significant amounts of subcutaneous fat and muscle deformation under its own weight. *Significance.* Our study is a robust characterization of angular deviations of muscle fiber directionality in the thigh as determined by DW-MRI. We show that an appropriate choice of experimental conditions reduces the variability of the observed directionality. Precise determination of tissue directionality will enable reproducible models of microscopic tumor spread, with future application in defining the clinical target volume for soft tissue sarcoma.

## Introduction

In recent years, the use of MRI in radiotherapy treatment planning has expanded tremendously. It is widely believed that it would be desirable to replace ionizing radiation-based CT planning scans with MRI. In addition, more accurate radiotherapy target delineation with MRI guidance may lead to improved treatment outcomes in a variety of cancers (Owrangi *et al* 2018). The addition of diffusion-weighted MRI (DW-MRI) to morphologic MR imaging is of great value in many clinical settings (Assaf *et al* 2013). The integration of DW-MRI with radiotherapy targeting has recently been demonstrated in glioma (Jordan *et al* 2019). The authors used white matter fiber tracking to calculate an anisotropic pathlength map that was translated into the radiotherapy target boundary. The anisotropic boundary was shown to be more consistent with the location of tumor recurrence (Jordan *et al* 2019). In prostate cancer, a recent pilot study has shown that information derived from DW-MRI can be used to characterize tumor tissue at both the macroscopic and microscopic levels for more precise localization of cancerous tissue (Langbein *et al* 2021). The value of DW-MRI for use in soft tissue radiation oncology is yet to be discovered and is likely to show similar benefits.

Microscopic studies confirm that tumor cells invade soft tissue along muscle fiber interfaces (Weigelin *et al* 2012). Based on this, we presented scientific evidence for the utility of DW-MRI in defining the extent of microscopic tumor spread in sarcomas (Shusharina *et al* 2022). We leveraged the well-established characterization of tissue microstructure with DW-MRI (Budzik *et al* 2007, Lansdown *et al* 2007, Rockel and Noseworthy 2016, Damon *et al* 2017, Berry *et al* 2018) and derived information on soft tissue directionality based on the anisotropic diffusion of water molecules. We used this information to develop a model for microscopic tumor propagation by calculating the shortest path in anisotropic media directly from the DW-MRI data without mapping muscle fibers with tractography (Shusharina *et al* 2022).

The diffusion-encoded signal obtained with magnetic field gradients along multiple directions results in voxel-wise diffusion tensors (DTs) that encode directions of water diffusion as eigenvectors of the tensor. In muscle, the largest eigenvalue corresponds to the direction along the fibers (Damon *et al* 2002), and thus the eigenvector associated with the largest eigenvalue is the preferred direction of tumor spread. Therefore, the directional variability of the principal eigenvector has a direct impact on the accuracy of defining the boundary of microscopic tumor spread in models such as Shusharina *et al* (2022).

Many radiation oncology departments have chosen to have an MR scanner available for imaging the patient in the treatment position. Such a setup not only helps to minimize misalignment between diagnostic MR images and planning CT images, but also allows for better target definition due to consistent patient positioning. With our DTI-based microscopic tumor extent definition method, it will be possible to automatically define gross tumor volume and to model microscopic tumor extent for treatment planning in a single MR imaging session. The aim of the present study is to establish baseline parameters and protocols for DW-MR acquisition for future studies in sarcoma patients by quantifying the directional variability of the principal eigenvector of the water DT in soft tissue and investigating significant factors influencing the variability.

## Materials and methods

### Image acquisition

Ten healthy volunteers (seven men and four women, age 37 ± 9 years) participated in this study, which was approved by the Institutional Review Board of Massachusetts General Hospital. Written informed consent was obtained from each subject. Volunteers were scanned feet first using a 3T MRI system (Siemens, Magnetom Prisma, Siemens Healthcare, Erlangen, Germany) and an 18-channel torso phased array coil covering the left and right thighs. The imaging protocol consisted of (a) two high-resolution anatomical (spin-echo, SE) scans, T1- and T2-weighted; (b) a diffusion-weighted (DW) spin-echo-based scan using an echo planar (EP) acquisition with fat suppression. Anatomical and diffusion-weighted MRI scans were acquired in the axial plane.

The DW-MRI acquisition consisted of two *b*_0_ images with *b*_0_ = 50 s mm^−2^ and twelve diffusion-weighted images with *b* = 400 s mm^−2^ using 12 gradient directions. Spectral adiabatic inversion recovery (SPAIR) was used to suppress the fat signal. Twelve independent acquisitions were performed for each *b* = 400 s mm^−2^ diffusion gradient direction. The images without diffusion-weighting were independently acquired two times. T1- and T2-weighted acquisitions were used to match the anatomical location of the muscles in DW images.

### Data processing and analysis

The diffusion-weighted series were resampled to an isotropic voxel size of 1.25 × 1.25 × 1.25 mm^3^ as most of the images were acquired with this planar resolution. For each acquired DW-MRI scan, the DT was reconstructed from 12 diffusion-encoded gradient pulses using the imaging Python library DIPY (Garyfallidis *et al* 2014) with the tensor model of Basser *et al* (1994).

**Figure 1.**
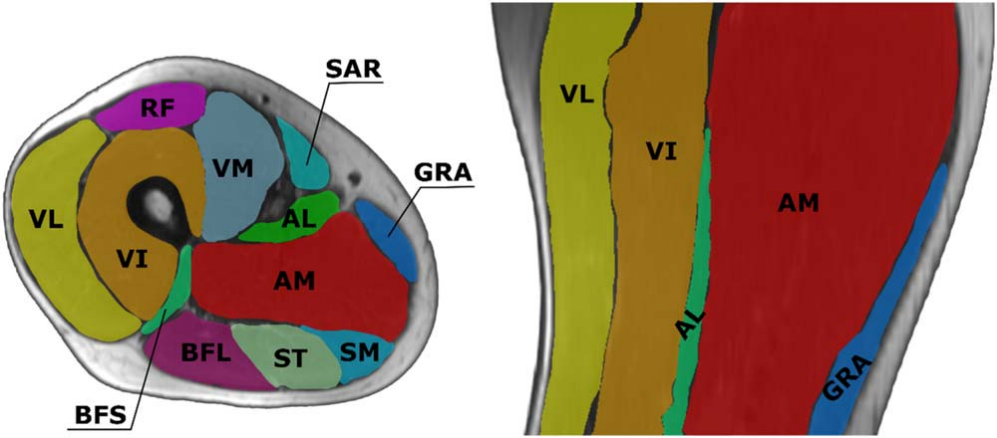
Representative T1-weighted MR anatomical image of the thigh, axial and sagittal views. The 12 muscles are: sartorius SAR, vastus medialis VM, vastus intermedius VI, vastus lateralis VL, rectus femoris RF, biceps femoris short head BFS, biceps femoris long head BFL, semitendinosus ST, gracilis GRA, semimembranosus SM, adductor longus AL, adductor magnus AM.

Twelve muscles of the tight were manually contoured on T1-weighted MR images of the left and right thigh (figure 1). The muscle masks were used to characterize the directional noise of the DT data within each of the 12 muscles. The noise was quantified as the voxel-wise angular deviation from the principal eigenvector averaged over all voxels within a muscle. Averaging was performed in the following steps. To remove data degeneracy due to up-down symmetry in the principal eigenvector direction, two clusters of vectors were detected using *k*-means and one of the two clusters was inverted by reflecting the vector direction, similar to Rockel and Noseworthy (2016). The mean vector was then calculated for each muscle. The voxel-wise angle between the eigenvectors, corrected for the reflection, and the average vector was then calculated.

### Experimental conditions

To define the optimal experimental conditions for directional stability, we compared data acquired with different coil configurations and subject immobilization, and examined the effect of subject position, supine versus prone. We also analyzed data obtained from subjects with different levels of subcutaneous fat.

## Results

### Histograms and maps of the principal eigenvector variability

Histograms of the principal eigenvector variability as angular deviation from the mean eigenvector within each muscle, of the left and right thigh were calculated for each subject. The mean modes of the histograms for each muscle are summarized in table 1.

**Table 1.** The mean mode of the diagram of the deviation angle from the average principal eigenvector calculated in each muscle of the thigh (left and right) for 10 subjects.

Muscle	GRA	VI	VL	AM	BFS	AL	RF	SAR	VM	SM	ST	BFL
Mean, deg	17.2	17.4	16.8	25.3	15	24	16.8	18.3	17.9	21.6	16.1	17.5
Std, deg	6.9	4.1	6.8	8	6.1	8.9	7.5	8.26	5.5	8	7.3	7.5

The experimental design was varied as follows. Subjects 1 through 9 were scanned in the supine position. Subject 1 was scanned with a flat coil covering each thigh in successive acquisitions. For this subject, one leg was immobilized by placing the calf in a cylinder so that the calf was elevated approximately 4 cm above the couch. In this setup, the thigh was partially suspended between the hip joint and the elevated calf. The other leg was scanned without immobilization. This position was somewhat uncomfortable for the subject, and other subjects were scanned without elevating the calf. For subjects 2 through 10, both thighs were scanned simultaneously with the coil covering both legs. Subject 10 was scanned in the prone position. The prone position was also found to be suboptimal for patient comfort. No leg immobilization was used for subjects 2 through 10.

**Figure 2.**
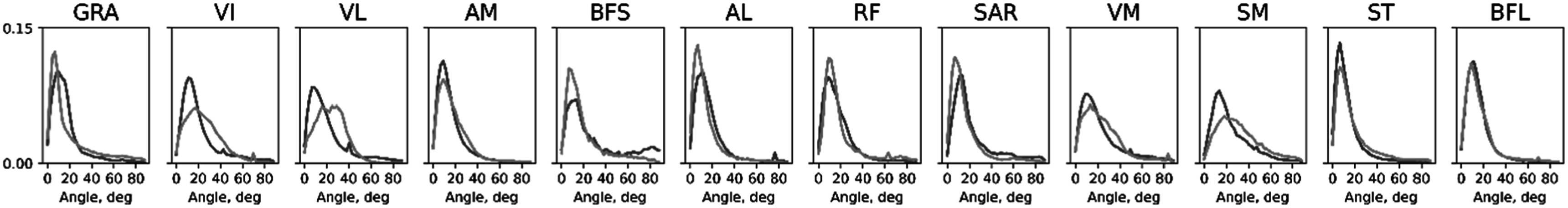
Histograms of the principal eigenvector variation in 12 muscles with consecutive scanning of the left and right thigh in the supine position; left calf was immobilized (blue) while right calf was not (orange).

The lowest variation of the angle of deviation was obtained in the case of the consecutive acquisition with the immobilized calf for a subject who was scanned in the supine position (see the histograms in figure 2). In this case, the lowest mode of the histogram was 6° (range: [6°–12°], with a standard deviation of 2.2°).

**Figure 3.**
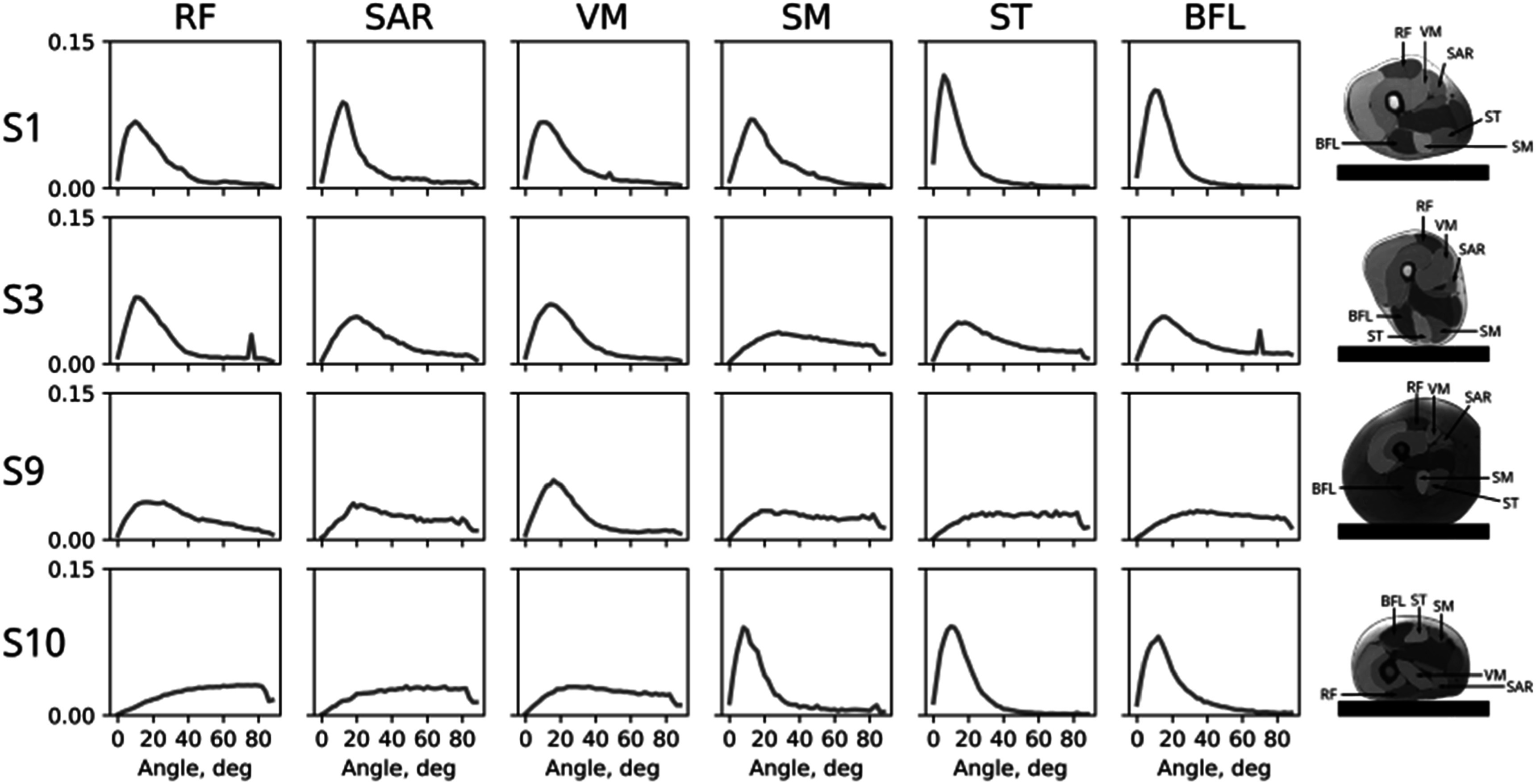
Histograms of principal eigenvector variability in six thigh muscles for four subjects scanned in the supine position, subjects S1, S3, S9, and one subject scanned in the prone position, S10. Subjects S3 and S9 differ in the amount of subcutaneous fat.

Figure 3 compares principal eigenvector variability in six muscles, three in the anterior thigh (rectus femoris (RF), vastus medialis (VM), and sartorius (SAR)) and three in the posterior thigh (biceps femoris long head (BFL), semitendinosus (ST), and semimembranosus (SM)) for four subjects. For the subject with an elevated thigh, the variation was nearly the same for the six muscles (S1 in figure 3). Variation was greater for muscles farther from the coil, posterior muscles for supine subjects (S3 and S9), and anterior muscles for the prone subject (S10). Variation was greater in a subject with thicker subcutaneous fat (S9). The greatest variation was observed in the front muscles that were pressed against the couch for the subject who was scanned in the prone position (S10).

**Figure 4.**
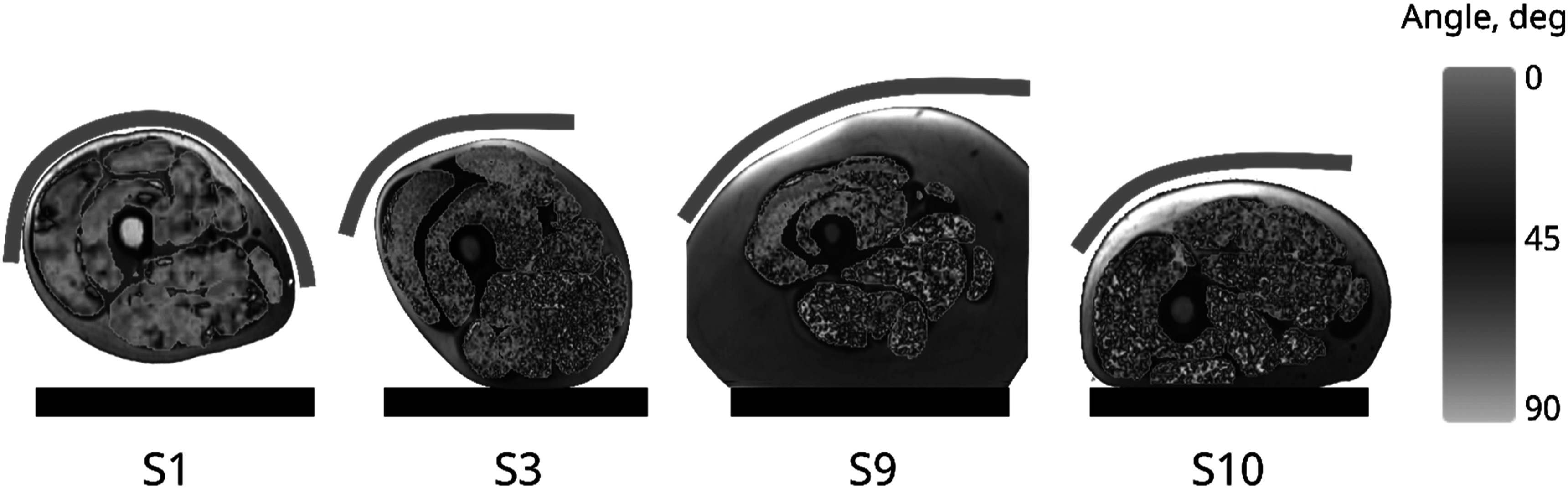
Principal eigenvector variability maps calculated within each muscle for the four subjects across experimental conditions. Subject 1 (S1) with consecutive scanning of the left and right thigh in the supine position with the calf immobilized; subjects 3 and 9 (S3 and S9) in the supine position with simultaneous acquisition using a flat coil covering both thighs; subject 10 (S10) in the prone position with simultaneous acquisition using a flat coil covering both thighs. The table and coil placement are shown schematically.

The maps of the deviation angles in the range from 0° to 90° show that the direction of the principal eigenvector changes gradually, with a higher degree of variation in the periphery of the muscles. Overall, the maps are consistent with the quantitative observations shown in figure 4. A higher degree of variability was observed in subjects with thicker subcutaneous fat and in muscles further away from the coil. The highest variability, especially in subjects scanned while lying prone, was observed in muscles deformed by pressing against the couch.

## Discussion

As DW-MRI makes its way into the clinic for radiotherapy targeting, it is important to quantify the accuracy and reproducibility of the method under real clinical acquisition conditions. Of all the DT parameters, we focus on the direction of the principal eigenvector, which is attributed to the direction of the muscle fibers (Damon *et al* 2002), and investigate its stability across muscles and experimental conditions. For the two minor eigenvectors, it has been shown that the assumption that these measures reflect anatomical structure may not be valid due to their high directional variability (Rockel and Noseworthy 2016).

Acquisition noise causes the DT eigenvalues and the direction of its principal axes to deviate from their true values. In our tumor propagation model (Shusharina *et al* 2022), the DW-MRI data were used to define the shortest path from the point inside a muscle to the distant points in the tissue. Both instrumental and biological noise will cause the shortest path isosurfaces to be less anisotropic and have a noisy structure. In this paper, we have quantified the directional noise in DW-MRI data acquired from 12 muscles of the human thigh. It was shown that the directional variability of the principal eigenvector of the voxel-wise DT can be as low as 6°. We have shown that with an appropriate experimental setup, including appropriate leg immobilization, the stability of the principal eigenvector can be improved. When the thigh was suspended with the flexible coil around it, the highest consistency of the vector was observed. Our study suggests that the directional stability of the principal eigenvector may be higher when a special limb MRI coil is used instead of the flat coil used in the present study.

Previous studies were focusing on reproducibility of the measures derived from DT (eigenvalues, fractional anisotropy and mean diffusivity) and fiber characteristics calculated from tractography (fiber length, and pennation angle) to assess feasibility of longitudinal studies of varying muscle conditions (Froeling *et al* 2010, Heemskerk *et al* 2010, Sinha and Sinha 2011). It was concluded that DW-MRI data is generally reproducible and varies across regions of interest and image acquisition parameters. The effect of acquisition parameters on the stability of the principal eigenvector was recently reported (Rockel and Noseworthy 2016). It was shown that stability can be improved by increasing the number of diffusion directions. In future studies we will focus on optimizing parameters to reach high reproducibility of DW-MRI data within clinically acceptable scan time.

Inaccurate estimation of the principal eigenvector may be caused by chemical shift artifacts in fat infiltrated muscle tissue within voxels containing both muscle fiber and fat. This is expected to be a problem in elderly patients and patients with muscular dystrophy. In addition, special consideration should be given to STS subtypes derived from adipose tissue, such as myxoid liposarcoma. Although it is accepted that SPAIR and STIR fat suppression techniques are optimal for the best performance of DW-MRI sequences (Guirguis *et al* 2022), as they effectively suppress aliphatic fat signals, additional effort may be required to suppress olefinic fat signal by applying the method based on time-shifted echo acquisition (Hernando *et al* 2011).

Subject motion can cause image artifacts and associated errors in DT reconstruction. The echo-planar imaging acquisition mode typical of DW-MRI allows acquisition of slices in less than 100 ms and is therefore less sensitive to bulk motion. To further reduce motion artifacts, faster acquisition modes (Wang *et al* 2018) and motion-limiting positioning can be used. Positioning must be reproducible for both imaging and treatment sessions, yet comfortable for the cancer patient, such as a scaffold system (Froeling *et al* 2010). The individual patient scaffold system would also be preferable because it allows suspended limb placement and can be used in the pre-treatment MR session and during radiation therapy.

## Conclusions

This study demonstrates that the spatial variability of the principal eigenvector in DW-MRI can be reduced by an appropriate choice of experimental conditions. We show that the variability of the observed directionality can be reduced by positioning the patient with the leg immobilized and the coil covering the entire thigh. This in turn leads to highly reproducible models of microscopic tumor spread. Future applications include the definition of clinical target volumes for soft tissue sarcomas.

## Data Availability

The data cannot be made publicly available upon publication due to legal restrictions preventing unrestricted public distribution. The data that support the findings of this study are available upon reasonable request from the authors.

## Ethical statement

The study involving imaging of healthy subjects was approved by the Institutional Review Board of Massachusetts General Hospital #2019P001781.
